# Robust performance of a live bacterial therapeutic chassis lacking the colibactin gene cluster

**DOI:** 10.1371/journal.pone.0280499

**Published:** 2023-02-02

**Authors:** Aida Kalantari, Michael J. James, Lauren A. Renaud, Mylene Perreault, Catherine E. Monahan, Mary N. McDonald, David L. Hava, Vincent M. Isabella

**Affiliations:** Synlogic, Inc., Cambridge, Massachusetts, United States of America; Western Michigan University, UNITED STATES

## Abstract

*E*. *coli* Nissle (EcN) is a non-pathogenic probiotic bacterium of the *Enterobacteriaceae* family that has been used for over a century to promote general gut health. Despite the history of safe usage of EcN, concerns have been raised regarding the presence of the *pks* gene cluster, encoding the genotoxin colibactin, due to its association with colorectal cancer. Here, we sought to determine the effect of *pks* island removal on the *in vitro* and *in vivo* robustness and activity of EcN and EcN-derived strains. A deletion of the *pks* island (*Δpks*) was constructed in wild type and engineered strains of EcN using lambda red recombineering. Mass spectrometric measurement of N-myristoyl-D-asparagine, released during colibactin maturation, confirmed that the *pks* deletion abrogated colibactin production. Growth curves were comparable between *Δpks* strains and their isogenic parents, and wild type EcN displayed no competitive advantage to the *Δpks* strain in mixed culture. Deletion of *pks* also had no effect on the activity of strains engineered to degrade phenylalanine (SYNB1618 and SYNB1934) or oxalate (SYNB8802). Furthermore, 1:1 mixed dosing of wild type and *Δpks* EcN in preclinical mouse and nonhuman primate models demonstrated no competitive disadvantage for the *Δpks* strain with regards to transit time or colonization. Importantly, there was no significant difference on *in vivo* strain performance between the clinical-stage strain SYNB1934 and its isogenic *Δpks* variant with regards to recovery of the quantitative strain-specific biomarkers d5- *trans*-cinnamic acid, and d5-hippuric acid. Taken together, these data support that the *pks* island is dispensable for Synthetic Biotic fitness and activity *in vivo* and that its removal from engineered strains of EcN will not have a deleterious effect on strain efficacy.

## Introduction

Polyketide synthases (*pks*), found in bacteria, fungi, and plants, synthesize a wide array of natural products of potential value, including some that possess antibiotic and immunomodulatory properties [1,2]. Conversely, some *pks*-derived products have been suggested to negatively impact human health. The colibactin *pks* gene cluster is a 54-kb genomic island containing 19 contiguous genes, starting with *clbA* and ending with *clbS*, that is expressed in enterobacteria, including select strains of *Escherichia coli* and *Klebsiella pneumonia* [3]. Colibactin is a genotoxin that can induce DNA damage in cell lines and pre-clinical models and is suggested to play a role in the pathogenesis of a subset of colorectal cancer (CRC) cases [4,5]. However, *E*. *coli* strains and other gut microbiota members that harbor the *pks* genomic island are not always known pathogens or identified solely in disease states. Indeed, *pks*^*+*^ bacteria have also been identified as commensal strains in healthy individuals [6]. One member of the *Enterobacteriaceae* family that possesses the *pks* gene cluster is the well-studied probiotic bacterium, *Escherichia coli* Nissle 1917 (EcN). For over a century, this gram-negative strain has been used to treat various gastrointestinal disorders, including inflammatory bowel disease and irritable bowel syndrome [2]. EcN is thought to interact with the intestinal epithelium in a manner that stimulates anti-inflammatory activities and helps to maintain barrier function [7]. Notably, after oral administration, EcN does not colonize humans long-term [8]. Despite the available literature on the safety of EcN as a probiotic strain, the presence of the *pks* gene cluster has raised concerns over its potential role in disease promotion.

A number of studies have demonstrated the potential for colibactin genotoxicity, although the direct translation of many of these findings to humans remains to be fully elucidated. *In vitro*, transient infection of eukaryotic cells with *pks*^*+*^ *E*. *coli* induced cell cycle arrest, DNA double-strand breaks, and chromosomal abnormalities, while *ex vivo* infection of colonic loops with *pks*^*+*^ *E*. *coli* in healthy mice resulted in significantly increased DNA damage to colonic epithelial cells [5,9]. Additionally, repeat luminal injection of human intestinal organoids with *pks*^*+*^ *E*. *coli* resulted in DNA damage and single-base substitutions, and these findings were absent in *pks*^*-*^ *E*. *coli*-treated organoids [5]. Furthermore, in a recent study, Nougayrede *et*. *al* reported DNA damage in the intestinal epithelial cells of mice dosed with EcN; though this damage was not present when animals were dosed with an isogenic EcN strain in which the *clbA* and *clbP* genes were removed [10]. However, it is important to note that these studies utilized mouse models that did not contain an intact microbiota and were essentially monocolonized with EcN. Indeed, in more physiologically relevant *in vivo* models, utilizing naïve rodents with intact microbiota, EcN displayed no deleterious effects *in vivo* [11], although the methodology used may not detect DNA damage with exposure to low levels of colibactin [12].

Several studies have provided evidence for a potential association between the presence of *pks*^*+*^ *E*. *coli* and CRC. Independent studies have reported the prevalence of *pks*^*+*^ *E*. *coli* in the fecal samples of healthy adults ranging from 24% to 32% and in healthy infants from 15% to 18% in the US and Europe [9,13,14]. In the same geographic regions, the prevalence of *pks*^*+*^ *E*. *coli* increases to 60% in patients with familial adenomatous polyposis (FAP) or CRC [3,5]. However, in contrast to these findings, a Japanese study reported a similar prevalence of *pks*^+^ *E*. *coli* in CRC patients (43%) and controls (46%) [15]. Preclinical studies characterizing the relationship between *pks*^*+*^ strains and CRC are also conflicting, but data suggests that *pks*^*+*^ *E*. *coli* do not spontaneously induce colorectal cancer without an additional predisposition or risk factor. For example, in one study, the presence of *pks*^+^ *E*. *coli* accelerated the progression from dysplasia to invasive carcinoma in AOM/ IL-10^-/-^, but not in wild-type mice [16]. In line with these observations, it has been reported that an intact mucus layer attenuates the genotoxic effect of colibactin [17]. The collective data suggest that long-term *pks*^+^ *E*. *coli* exposure is potentially associated with CRC in the context of dysbiosis, inflammation, and/or an impaired mucosal barrier. However, more data is needed to understand the causality and the magnitude of the clinical risk of colibactin in the setting of the complex human colonic microbiome.

Live biotherapeutic strains of bacteria based on EcN, referred to as Synthetic Biotics, are advancing to clinical evaluation [18–20], several of which may be used as chronic, long-term therapies. An engineered EcN strain with a deletion in the *pks* island and that is unable to produce colibactin, would significantly reduce potential risks associated with colibactin production in man. To this end, we have constructed strains of *Δpks* EcN in several genetic backgrounds and characterized the effect of *pks* deletion on the growth, transit, and colonization phenotypes on EcN-derived strains. Additionally, the activity of recombinant genetic circuits when expressed in *Δpks* EcN strains was compared with wild type EcN. Our results demonstrate robust performance of engineered EcN following removal of the *pks* island both *in vitro* and *in vivo*. The results presented here may be helpful for the development of future live biotherapeutics to mitigate the potential risks associated with colibactin.

## Materials and methods

### Strain construction

In this study, *Escherichia coli* Nissle 1917 (EcN) from the German Collection of Microorganisms and Cell Cultures (DSMZ Braunschweig, *E*. *coli* DSM 6601) was used. Deletion of the colibactin-producing loci from the EcN genome was performed using phage lambda-derived red recombination system [21].

First, the following primers:

ATATCTATTGCTCCTTGTATAGTTACACAACTATTTATTACACGTCTTGAGCGAT (KO FW) and TTATCGACGGCTCAGAAGTGTCTAGATTATCCGTGGCGATCTGACATGGGAATTAGCCA (KO REV) were used in a PCR reaction with PKD4 plasmid as a template, to generate a fragment containing chromosomal homology to the *clb* loci. The fragment contained a kanamycin resistance gene flanked by FRT (flippase recognition target) sites. Next, EcN containing PKD46 was transformed with the *Δpks* fragment by electroporation. Transformed cells were selected as colonies on Lysogeny Broth (LB) agar plates containing kanamycin at 50 μg/ml. Accuracy of the obtained KO colonies was further confirmed both with PCR (FW: ATATCTATTGCTCCTTGTATAGTTACACAACTATTTATTACACGTCTTGAGCGAT and REV: CCGCATTGCATCAGCCATGAT) and Sanger sequencing. This method was used to remove the *pks* region from WT EcN, SYNB1618 [19], SYNB1934 [22], and SYNB8802 [20], resulting in *Δpks* EcN, *Δpks* SYNB1618, *Δpks* SYNB1934, and *Δpks* SYNB8802, respectively (Table 1).

**Table 1:** Strains list.

Strain	Antibiotic resistance	Genotype	Accession number
WT EcN	Streptomycin	*Escherichia coli* Nissle 1917	n.a.
*Δpks* EcN	Kanamycin	*Escherichia coli* Nissle 1917: ΔclbA-clbQ*	n.a
SYNB1618	None	*Escherichia coli* Nissle 1917: *ΔdapA*, Δprophage, *ΔlacZ*, *lacZ*::P_*fnrS*_-*pheP*, *agaI/rsmI*::P_*fnrS*_-*pheP*, *araBC*::P_*BAD*_*-*LAAD, *malEK*::P_*fnrS*_*-*PAL, *malPT*::P_*fnrS*_-PAL, *yicS/nepI*::P_*fnrS*_-PAL, *exo/cea*::*lacI-*P_*tac*_-PAL, *rhtBC*::*lacI-*P_*tac*_-PAL	PRJNA482064
*Δpks* SYNB1618	None	*Escherichia coli* Nissle 1917: *ΔdapA*, Δprophage, *ΔlacZ*, *lacZ*::_P*fnrS*_-*pheP*, *agaI/rsmI*::P_*fnrS*_-*pheP*, *araBC*::P_*BAD*_*-*LAAD, *malEK*::P_*fnrS*_*-*PAL, *malPT*::P_*fnrS*_-PAL, *yicS/nepI*::P_*fnrS*_-PAL, *exo/cea*::*lacI-*P_*tac*_-PAL, *rhtBC*::*lacI-*P_*tac*_-PAL, *ΔclbA-clbQ*	n.a
SYNB1934	None	*Escherichia coli* Nissle 1917, *ΔdapA*, Δprophage, *rhtB/C*::*lacI*-P_*tac*_-*pheP*, *araBC*::*P*_*BAD*_*-*LAAD, *malK/phage tail protein*::*lacI-P*_*tac*_*-*mPAL, *yicS/nepI*::*lacI*-P_*tac*_-mPAL, *aga/rsmI*::*lacI*-P_*tac*_-mPAL, *exo/cea*::*lacI*-P_*tac*_-mPAL	PRJNA749270
*Δpks* SYNB1934	None	*Escherichia coli* Nissle 1917, *ΔdapA*, Δprophage, *rhtB/C*::*lacI*-P_*tac*_-*pheP*, *araBC*::*P*_*BAD*_*-*LAAD, *malK/phage tail protein*::*lacI-P*_*tac*_*-*mPAL, *yicS/nepI*::*lacI*-P_*tac*_-mPAL, *aga/rsmI*::*lacI*-P_*tac*_-mPAL, *exo/cea*::*lacI*-P_*tac*_-mPAL, *ΔclbA-clbQ*	n.a
SYNB8802	None	*Escherichia coli* Nissle 1917, *ΔthyA*, Δprophage, *agaI/rsmI*:: P_*fnrS*_−*scaaE3-oxdc-frc*, *exo/cea*:: P_*fnrS*_-*oxlT*	CP087958
*Δpks* SYNB8802	None	*Escherichia coli* Nissle 1917, *ΔthyA*, Δprophage, *agaI/rsmI*:: P_*fnrS*_-*scaaE3-oxdc-frc*, *exo/cea*:: P_*fnrS*_-*oxlT*, *ΔclbA-clbQ*	n.a

List of strains used and their relevant antibiotic resistance (where applicable).

*Δpks = ΔclbA-clbQ. Gene names separated by “/” designate the genomic regions flanking the insertion of the indicated gene.

In order to make streptomycin resistant EcN, cells were grown overnight in 5ml LB. On the following day, cells were back diluted in 25 ml LB (1:1000) and incubated in a 37^○^C orbital shaker (250 rpm), for 8 hours. The culture was centrifuged at 8000 rpm for 10 minutes. Cells were resuspended in 200 μl LB and 100 μl was spread on an LB + streptomycin 300 ug/ml plate and incubated at 37^○^C overnight. Several colonies that emerged from this plate were streaked on fresh LB + streptomycin 300 ug/mL plates and incubated at 37^○^C overnight. Several colonies were inoculated overnight in 3 ml LB media + Streptomycin 300 ug/mL. The *rpsL* loci was amplified using forward primer: CGCCCTAAAATTCGGCGTCC and reverse primer: CTGACCAATGACGCGACGAC using the liquid culture as template DNA. Amplicons were sequenced and the *rpsL* mutation was confirmed by sequencing.

### Growth curves of WT EcN and *Δpks* EcN strains

WT EcN and *Δpks* EcN strains (Table 1) were cultured individually overnight in LB broth or M9 minimal media (M9), 3mL at 37°C in 14 mL culture tubes. FeSO4 was added to the cells grown in M9 in the following three concentrations: 0μM, 100μM and 200 μM. Overnight cultures were diluted 1:100 in LB or M9 and grown to early logarithmic phase. Once the cell OD_600_ reached approximately 1, seed cultures were used to inoculate 150μL (individually or in 1:1 ratio) of relevant media. The OD_600_ of the bacterial culture was continuously monitored for 24h using a 96-well plate reader (Biotek, USA), at 37°C, with three biological replicates. For LB-grown cells, in a parallel experiment, the same procedure was followed and samples from each culture at different timepoints (0, 3, 6, 24h) were plated on LB agar or LB agar containing antibiotics (streptomycin 300μg/mL, kanamycin 100μg/mL) where relevant, to enumerate colony forming units (CFUs).

### Colibactin biomarker (N-myristoyl-D-Asparagine) detection

N-myristoyl-D-Asparagine was quantitated in bacterial supernatant by liquid chromatography-tandem mass spectrometry (LC-MS/MS) using a ThermoVanquish UHPLC-Altis TSQ MS system. Standard was prepared at concentrations from 0.032 to 20 μg/mL in 80% acetonitrile. Cells were cultured overnight in 3mL LB media (_+_ appropriate antibiotics) in a 15mL falcon tune, at 37F0B0C with shaking at 7 g. Supernatants were harvested from overnight cultures by centrifugation for 1 min, 11200g. Samples were then extracted with acetonitrile to 80%. Two microliters were injected onto a Thermo Hypersil Gold 5μM C18, 2.1 x 100 mm column using 0.1% formic acid (A) and acetonitrile with 0.1% formic acid (B) at 0.4 mL/min and 40°C. Analytes were separated using a gradient from 30 to 98% B over 2 min followed by wash and equilibration steps. Compounds were detected by tandem mass spectroscopy with selected reaction monitoring in electrospray negative ion mode using the N-myristoyl-D-asparagine specific collision induced mass transitions: 343/211, 343/326, 343/133. Peak areas of the 343/211 ion pair were used to calculate unknown concentrations against the standard calibration curve. Lower limit of quantitation (LLOQ) was 0.8μg/mL.

### TCA assay

In order to determine Phe metabolizing activity *in vitro*, PKU strains (Table 1) were grown to exponential phase, then IPTG was added at 1 mM (final concentration) to induce expression of phenylalanine ammonia lyase (PAL). After 18h cells were centrifuged for 10 min at 5000 × g and pellets were resuspended in Formulation Buffer (100 mM potassium phosphate + 25% glycerol) and frozen at -80°C until testing. Frozen cell aliquots were thawed, diluted to an OD_600_ of 1 in assay buffer (M9 minimal media containing 0.5% glucose and 40mM L-Phenylalanine) to a final volume of 1mL, and then incubated without shaking at 37°C for 2h in a 96-deep well plate. At 0, 30, 60, 90, and 120 min, 150μL of the samples were collected in a v-bottom 96-well plate and centrifuged at 1792 g for 2 min. Aliquots of the supernatants (100μL) were transferred to a clear flat-bottom 96-well plate for TCA measurement at 290 nm absorbance. TCA standards were prepared in the assay buffer with the following concentrations: 0.625, 0.313, 0.156, 0.078, 0.039, 0.02, and 0.01mM. A standard curve was generated with absorbance values at 290 nm for each of the concentrations and was used to convert absorbance values to mM TCA produced during the assay. TCA production rates were calculated using the quantity of TCA calculated for each of the 0-, 30-, 60-, 90-, and 120-min time points, and normalized by 1e9 cells/mL (assuming OD_600_ of 1 = 1e9 cells) per hour. Final TCA production values were reported as μmol/hr/1e9 cells.

### Oxalate degradation assay

To measure oxalate metabolizing activity, HOX strains (Table 1) were grown overnight, diluted 1:100 and grown in shake flasks for 2h at 37^○^C under aerobic conditions, and subsequently transferred in an anaerobic chamber for 4 h to induce expression of the oxalate metabolizing pathway. Induced cells were collected by centrifugation (5000 × g, 10 min), followed by concentration and freezing at ≤ -65°C in glycerol-based formulation buffer (PBS + 25% glycerol). Activated cells were resuspended to OD_600_ = 5 in assay media containing 10 mM oxalate and incubated statically at 37°C. Supernatant samples were removed at 30 and 60 min to determine the concentrations of oxalate using a liquid chromatographic (Waters Acquity UPLC® system controlled by Empower 3 (build 3471) or greater) separation with ultraviolet (215 nm) detection. The results are interpreted against an oxalate standard curve to ascertain quantification. The activity is defined as the amount of oxalate consumed (difference between the T = 0 and T = 60 min), as expressed as μmol oxalate/hour/1e9 cells.

### Ethical statement

All procedures performed on animals were in accordance with the humane guidelines for ethical and sensitive care by the Institutional Animal Care and Use Committee (IACUC) of the U.S. National Institutes of Health. Procedures and protocols related to mouse studies were reviewed and approved by Mispro Biotech Services’ Institutional Animal Care and Use Committee. Standard operating procedures related to Nonhuman Primate (NHP) studies have been reviewed and approved by Charles River Laboratories’ Institutional Animal Care and Use Committee.

### Mouse excretion studies

C57BL/6J mice (n = 10; The Jackson Laboratory, ME) of approximately 20–22 weeks of age were group housed, weighed, and assigned to groups based on body weight (n = 5/group). In the first set of experiment, mice received a single oral dose (1x10^10^ CFUs) of WT EcN (streptomycin resistant) or *Δpks* EcN (kanamycin resistant) and fecal pellets were collected fresh by free catch for up to 48h. A second set of experiments was designed in which the resident microbiota was altered using streptomycin, a condition conducive for longer-term EcN colonization. Two independent studies were conducted and the data from both studies were combined. Mice were provided with streptomycin (5 g/L) in their drinking water for 24h before they received a single combined oral dose (1x10^10^ CFU of each bacterial strain) of WT EcN (streptomycin resistant) and *Δpks* EcN (streptomycin and kanamycin resistant). Fecal pellets were collected fresh by free catch for up to 72h. At the end of the studies, mice were euthanized by CO_2_ asphyxiation.

At each timepoint, feces were placed into pre-weighed bead-bug tubes, weighed, 500 μL of PBS was added, and samples were processed for serial dilution. Homogenates were plated in duplicate on agar containing antibiotics (streptomycin 300 μg/mL or kanamycin 100 μg/mL) selective for each strain to determine viable CFUs immediately after collection. Plates were incubated overnight at 37°C, and CFUs were counted the following day and normalized to fecal pellet weight. When no colonies were recovered, the presence of one CFU was assumed and corrected by the lowest dilution factor, and that value was used in the mean calculation. Using this approach, the lower limit of quantification was 1.5 x 10^2^ CFU/g feces.

### NHP studies

#### Husbandry and monitoring

Animals were socially housed (except during designated procedures) in wire mesh floor cages with automatic watering system at 64–84°F (18–29°C) with 30–70% humidity and 12 hours:12 hours light:dark cycle and provided items such as perches, floor toys, foraging/hanging devices. Additional enrichment like music, natural sounds or color videos were also provided. Animals were fed PMI Nutrition International Certified Primate Chow No. 5048 twice daily (except during designated procedures) in amounts appropriate for the size and age of the animals and offered food supplements (treats and/or fresh fruits). Animals were observed for moribundity/mortality twice daily (morning and afternoon) and for clinical signs at each scheduled timepoint. Veterinary care was provided throughout the course of the study and animals were examined by the veterinary staff as warranted by clinical signs or other changes. At the end of each study, animals were returned to the colony and allowed a 7-day wash-out period before being re-enrolled into the next experiment.

#### Single dose excretion study

Twelve non-naïve male cynomolgus monkeys (2–5 years of age) were group housed in wire-mesh floor cages, except for collection time points during study, and maintained in a controlled temperature and humidity environment (12-hour light/dark cycle) with water ad libitum and food provided in amounts appropriate for the size and age of the animals twice daily. On the morning of the experiment, monkeys received a single oral dose of a combination of WT EcN (streptomycin resistant) and *Δpks* EcN (kanamycin resistant) (1x10^12^ CFUs of each bacterial strain) with sodium bicarbonate (1.8 mmol), and feces were collected for up to 120h (n = 6 per group). Fecal samples were homogenized in bead-bug tubes containing 1mL of PBS and processed for serial dilution. Homogenates were plated in triplicate on agar containing antibiotics selective (streptomycin 300 μg/mL or kanamycin 100 μg/mL) for each strain to determine viable CFUs immediately after collection. Plates were incubated overnight at 37°C, and CFUs were counted the following day and normalized to fecal pellet weight. When no colonies were recovered, the presence of one CFU was assumed and corrected by the lowest dilution factor, and that value was used in the mean calculation. Using this approach, the lower limit of quantification was 1.5 x 10^2^ CFU/g feces.

*EcN strain activity*. Twelve non-naïve male cynomolgus monkeys were fasted overnight and orally administered peptone (3.05 g) followed by sodium bicarbonate (1.8 mmol), d5-Phe (154 mg) and SYNB1934 or *Δpks* SYNB1934 (1x10^11^ live cells). The study was conducted in a cross-over manner so that all 12 animals received each treatment following one week washout between each arm. Plasma was collected at 0-, 0.5-, 1-, 2-, 4- and 6-hours post-dose for d5-TCA measurements and cumulative urine was collected at 6h for d5-HA recovery (and normalized to creatinine levels to account for differences in urinary volumes). Because of significant reflux noted in one animal in the *Δpks* SYNB1934 group at the time of dosing, the data from this animal was excluded from further analysis. For samples measuring levels below the lower limit of quantification (LLOQ), the half LLOQ value was used to calculate the metabolite concentration.

### Metabolite quantitation in NHP plasma or urine by LC-MS/MS

NHP plasma concentrations of d5-TCA, as well as d5-HA and creatinine in NHP urine were quantitated by LC-MS/MS using a Thermo Ultimate 3000 UHPLC- TSQ Quantum MS system. Standard solutions from 0.16 to 250μg/mL were made in water. Neat plasma, 40-fold diluted urine, and standards in water were derivatized with 50mM each of 2-hydrazinoquinoline, dipyridyl disulfide, and tripheylphospine in acetonitrile containing 1μg/mL ^13^C_9_^15^N-Phe and d5-creatinine as internal standards and incubated at 60°C for 1h. Following derivatization, 20μL of standards and samples were diluted with 180μL 0.1% formic acid / acetonitrile (140:40). Ten microliters were injected onto a Phenomenex Luna 5μm C18(2) 100A, 100 x 2 mm column using 0.1% formic acid (A) and acetonitrile with 0.1% formic acid at 0.5 mL/min after an initial 10% B hold for 0.5 minutes using a gradient from 10 to 97% B over 1.5 minutes followed by wash and equilibration steps. Compounds were detected by tandem mass spectroscopy with selected reaction monitoring in electrospray positive ion mode using the following ion pairs: d5-TCA 295/136, d5-HA 326/160, creatinine 114/44, d5-creatinine 119/49. Chromatograms are integrated and analyte/internal standard peak area ratios were used to calculate unknown concentrations.

## Results

### *E*. *coli* Nissle engineered with genomic deletion of *pks* does not produce colibactin

**Fig 1:**
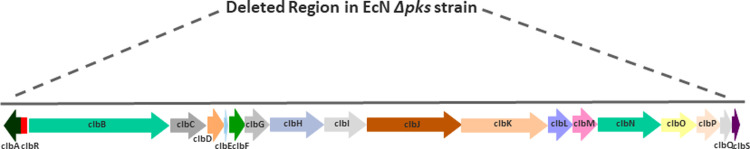
Schematic of an intact *pks* island coding for colibactin. This cluster consists of 19 genes starting with *clbA* and ending with *clbS*. The region of the *pks* island deleted is shown by dashed lines.

Many enterobacteria, including EcN, harbor the colibactin *pks* genomic region, which includes 19 contiguous genes starting with *clbA* and ending with *clbS* (Fig 1). To generate colibactin-deficient EcN strains, the genes in the *pks* island involved in colibactin production were deleted using lambda Red recombineering (Fig 1) [21]. The coding region of the *clbS* gene, whose product is involved in resistance to colibactin, was retained in the genome, however, the *clbS* promoter was removed. Genome wide transcriptomic data subsequently demonstrated that this was sufficient to prevent *clbS* expression (S1 Table).

**Fig 2:**
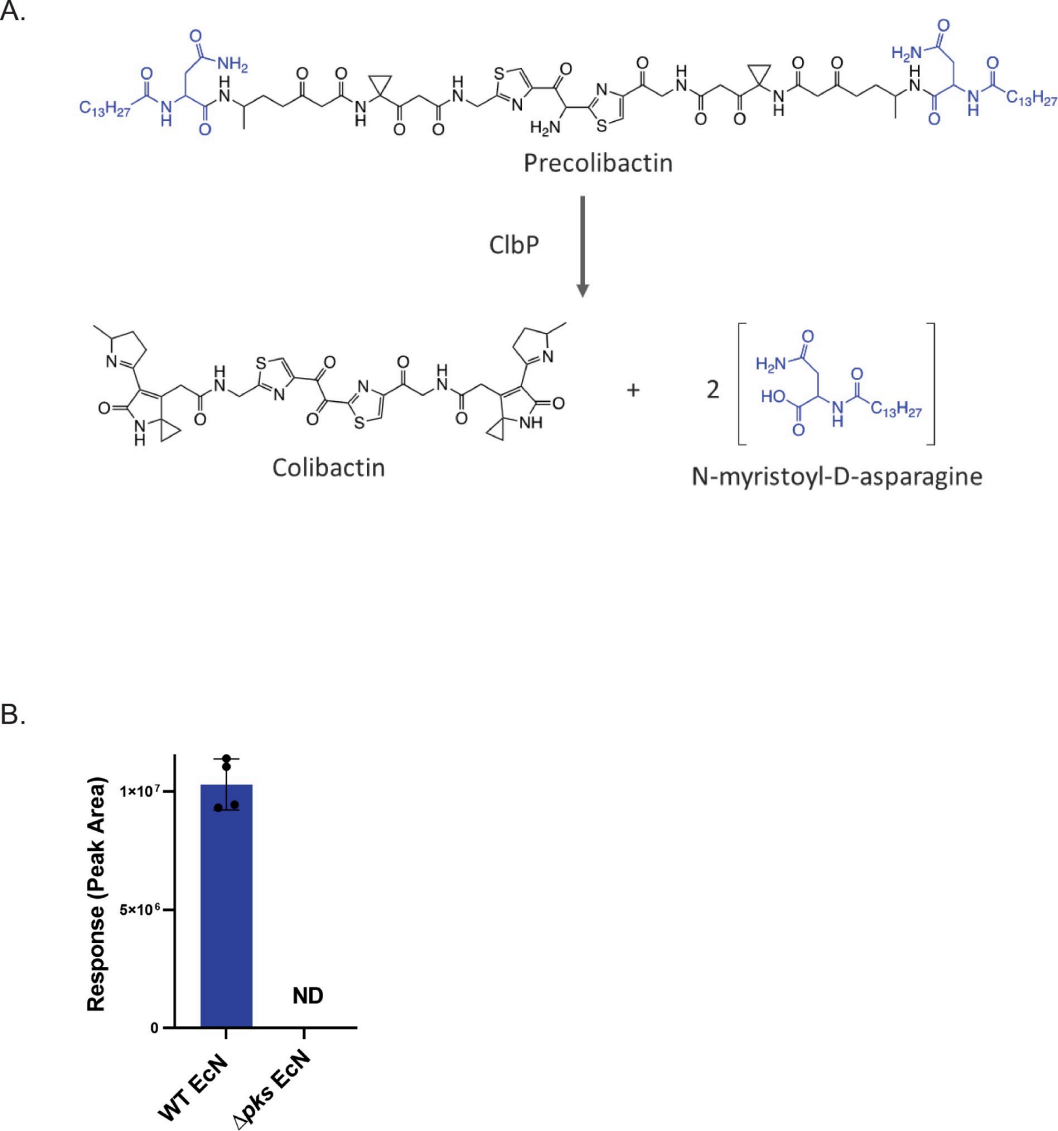
Δ*pks* EcN does not produce colibactin. A) The maturation of colibactin by ClbP results in two molecules of N-myristoyl-D-asparagine, which can subsequently be used as biomarker for colibactin production in EcN strains. B) N-myristoyl-D-asparagine is not detected in Δ*pks* EcN, while it is detected in WT EcN strain. ND, none detected.

Following the deletion of *pks* from EcN, we tested the ability of the strain to produce colibactin, which is a labile compound difficult to detect directly [23]. The structure of colibactin was only recently solved using a combination of approaches including multiple modifications to the *pks* genomic island, a number of analytical techniques, and chemical synthesis of proposed and later confirmed structures found within the colibactin biosynthetic pathway [24]. The colibactin pathway-specific small molecule N-myristoyl-D-asparagine has been identified in the literature; two molecules of this compound are produced during the conversion of colibactin precursor to mature colibactin [25], (Fig 2A). Detection and quantification of this metabolite has been used to characterize colibactin production in both EcN [26] and pathogenic *E*. *coli* cultures since it is more easily detected than measuring colibactin directly [27]. To assess colibactin production by wild-type EcN and the *Δpks* EcN strain, bacterial supernatants from overnight cultures were used for detection and quantification of this compound. Using this assay, N-myristoyl-D-asparagine was detected in the supernatants of WT EcN, but was below the limit of quantification in supernatants from the *Δpks* EcN strain (Fig 2B).

### Fitness and growth competition of WT EcN and *Δpks* EcN

**Fig 3:**
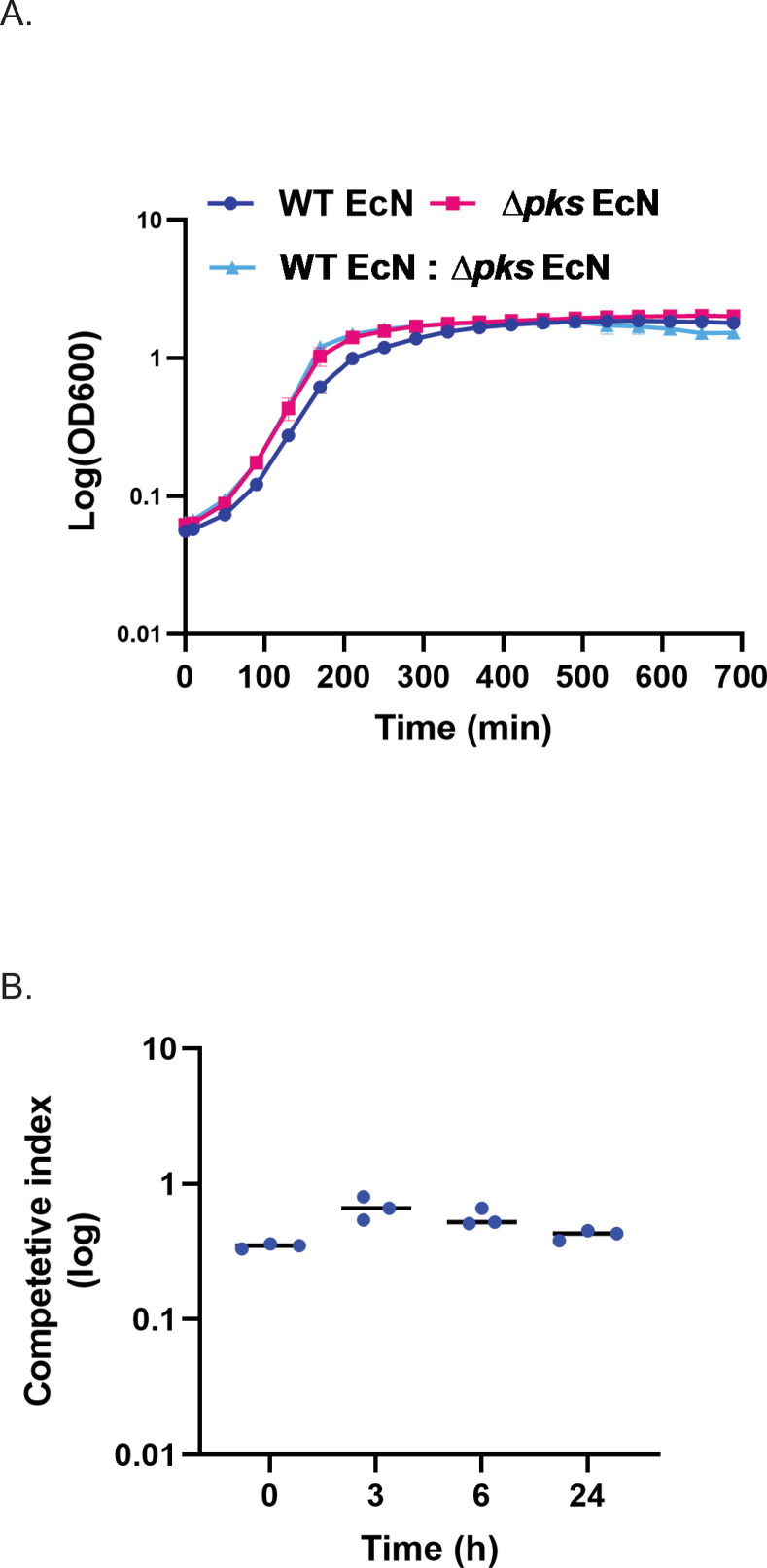
*In vitro* evaluation of EcN and *Δpks* EcN fitness. A) Growth curves of WT EcN (dark blue), *Δpks* EcN (pink) and both strains in mixed culture/competition (inoculated at a 1:1 ratio, light blue). Data are the mean and SD of triplicate cultures grown in LB media. B) Competitive index for *Δpks* EcN and WT EcN over a 24h period of growth in LB media. Competitive indexes were calculated at each time point by dividing the number of recovered *Δpks* EcN CFU (kanamycin resistant) by the number of recovered WT EcN CFU (streptomycin resistant). Each dot represents the CI determined from a single culture. A bar indicates the mean of the data. A competitive index of 1.0 indicates no difference in CFU recovery between the two strains.

Bacteria grown in LB broth cultures were used to evaluate the effect of *pks* island removal on cell fitness. Growth curves of three groups were compared: WT EcN, *Δpks* EcN, and a mixed culture of both strains inoculated at a 1:1 ratio. There were no gross differences in the growth curves of EcN, *Δpks* EcN, or the mixed culture, suggesting that removal of *pks* does not impact the rate or phases of growth (Fig 3A). Colibactin can be toxic to bacterial strains lacking a mechanism of resistance such as expression of *clbS*, an enzyme involved in inactivation of accumulated cytoplasmic colibactin [3]. The deletion of the *clbS* promoter in *Δpks* EcN, which resulted in loss of *clbS* expression, led to concerns that growth and survival of *Δpks* EcN could be compromised in the presence of a colibactin-producing WT EcN strain. However, WT EcN did not outcompete the *Δpks* EcN strain in mixed culture over a 24h period (Fig 3B). In total, deletion of *pks* does not appear to reduce cell growth or compromise survival in the presence of colibactin.

### Integrity of genetic circuit performance in the absence of *pks*

**Fig 4:**
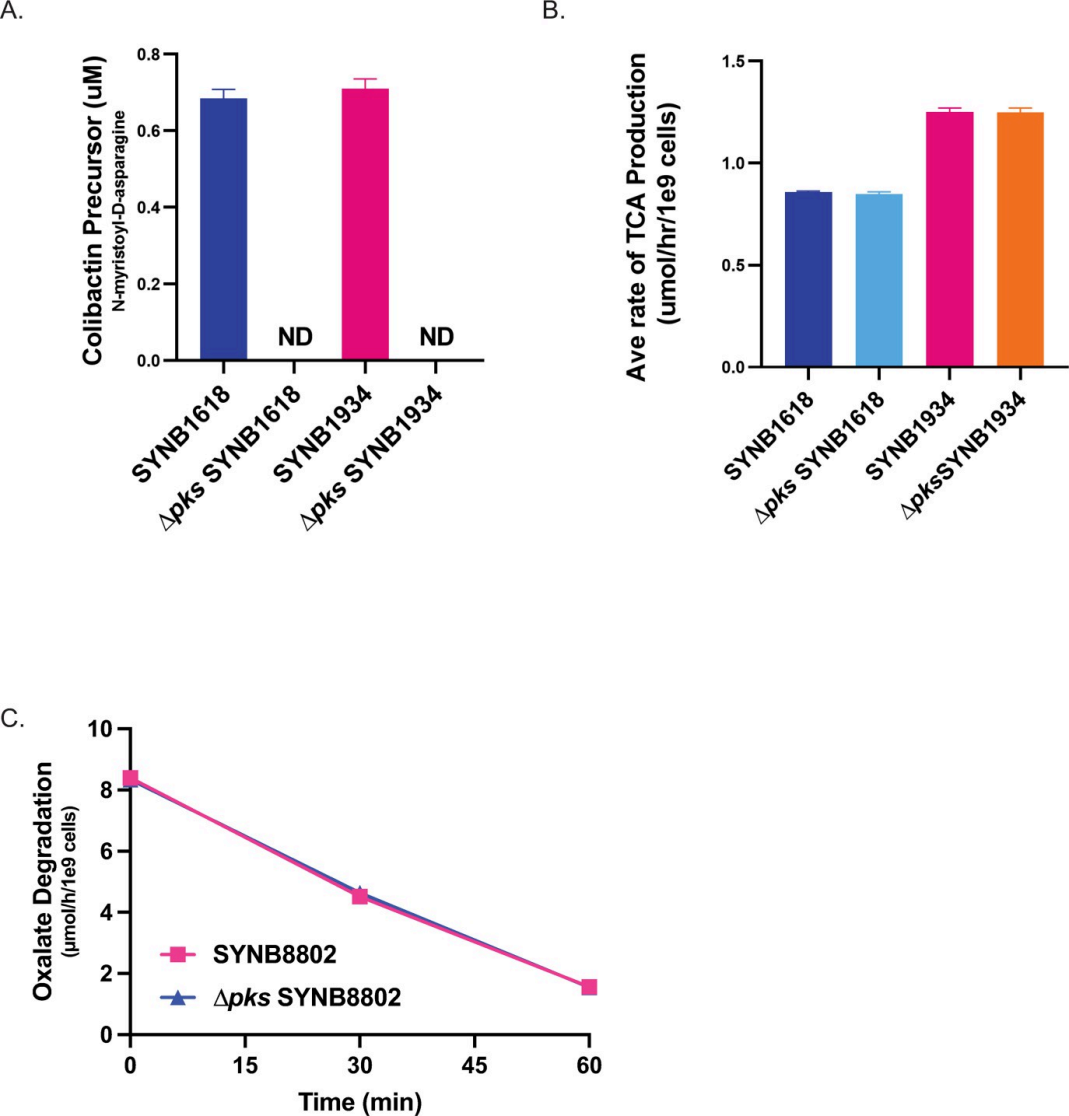
*In vitro* activity of engineered EcN strains in the absence of *pks*. A) N-myristoyl-D-asparagine (colibactin biomarker) levels in supernatants of parental strains SYNB1618 (dark blue) and SYNB1934 (pink), while no biomarker is detected in *Δpks* SYNB1618 and *Δpks* SYNB1934. Data are the mean ± SD of 3 samples after growth in LB for 18h. ND = not detected, (LLOQ) was 0.8μg/mL. B) SYNB1618 and SYNB1934 Phe metabolizing activity *in vitro*, before and after deletion of the *pks* island. Bar graph shows the mean ± SD (n = 3) rate of TCA production in SYNB1618 (dark blue), *Δpks* SYNB1618 (light blue), SYNB1934 (pink), and *Δpks* SYNB1934 (orange) using an *in vitro* assay of TCA production over 1h. C) SYNB8802 oxalate metabolism with and without the *pks* island. Oxalate metabolism by SYNB8802 (intact *pks* island; dark blue) and *Δpks* SYNB8802 (deleted *pks* island; pink) were measured over 1h. Mean ± SD of 3 samples at each time point are shown.

EcN is a chassis organism used for the construction of live biotherapeutic organisms indicated for the treatment of human disease. Two EcN-based live biotherapeutic strains, SYNB1618 and SYNB1934, have advanced to human clinical trials as potential treatments of phenylketonuria (PKU), a metabolic disease in which patients are unable to break down the amino acid Phe, leading to neurotoxicity. These strains have been genetically modified to express phenylalanine ammonia lyase (PAL), an enzyme that catabolizes Phe into the non-toxic metabolite *trans*-cinnamate (TCA). To determine if the deletion of *pks* resulted in any changes in the engineered function of these strains, the *pks* island was removed from both SYNB1618 and SYNB1934 and the functional deletion was confirmed by the absence of N-myristoyl-D-asparagine production in culture supernatants compared to parental strains (Fig 4A). We next sought to clarify whether there was any effect on the whole cell PAL activity of SYNB1618 or SYNB1934 in a *Δpks* background. Rates of TCA production from Phe were measured *in vitro* and demonstrated no significant difference in the *Δpks* strain backgrounds compared to their isogenic parents (Fig 4B). These results demonstrate that the ability to produce colibactin has no impact on the engineered activity of these strains. Another EcN-based live bacterial therapeutic strain, SYNB8802, is in clinical testing as a potential treatment of enteric hyperoxaluria, a disease involving the hyperabsorption of oxalate that results in the recurrent formation of oxalate-containing kidney stones [20]. Similar to that observed for the PKU strains, SYNB8802 engineered with a deletion of *pks* resulted in an *in vitro* activity profile comparable to that of the isogenic parent strain (Fig 4C).

### Gastrointestinal transit and survival of *Δpks* EcN

**Fig 5:**
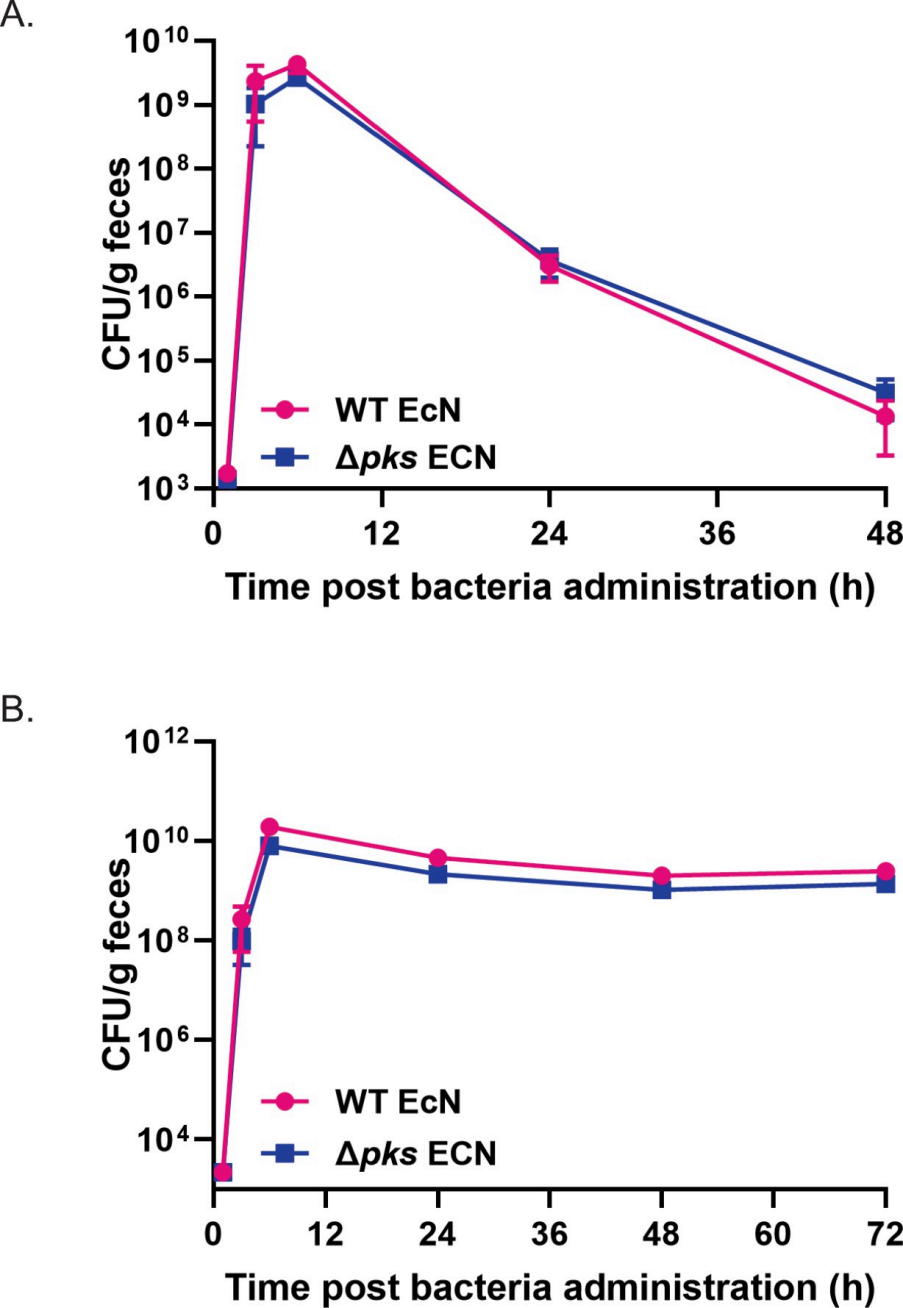
*In vivo* kinetics of WT EcN and *Δpks* EcN strains in mice. (A) Fecal excretion of EcN strains in C57BL/6J mice (n = 5 per group) following oral administration of a single dose (1x10^10^ CFU) of WT EcN (pink) or *Δpks* EcN (dark blue) over 48h. (B) Fecal excretion of EcN strains in streptomycin-treated C57BL/6J mice (n = 10 per group) following oral administration of a combined dose (5x10^9^ CFU of each bacterial strain) of WT EcN (pink) and *Δpks* EcN (dark blue) over 72h. In both cases, bacterial abundance was determined by CFU enumeration at each time point. Data are presented as the mean ± SEM. Statistical analysis was performed using 2-way repeated ANOVA followed by Sidak’s multiple comparison test, (p >0.05 for all time points).

Having demonstrated no deleterious impact to bacterial growth or activity *in vitro*, subsequent studies aimed to evaluate strain performance of *Δpks* EcN mutants *in vivo*. First, to compare bacterial cell transit and survival *in vivo*, WT EcN or *Δpks* EcN were orally administered to female C57BL/6J mice (n = 5 per group) at 1x10^10^ CFUs and collected by free catch for up to 48h after dosing. Fecal samples were homogenized in PBS and processed for quantification of bacteria by serial dilution plating, and CFU counts from fecal pellets were monitored over time to serve as relative indicators of survival and GI transit time. Fecal recovery of both WT EcN and *Δpks* EcN peaked at 6h post-bacteria administration, with 4.3 × 10^9^ and 2.7 × 10^9^ CFU/g feces, respectively, and the kinetics and clearance of both strains were not significantly different (Fig 5A). These data demonstrate that the presence of an intact microbiota in this mouse model does not compromise survival of *Δpks* EcN compared to its isogenic parent and further that neither the WT EcN or *Δpks* EcN strains colonize mice containing an intact microbiota, consistent with previously published reports [28].

In a subsequent mouse study, the resident microbiota was altered using streptomycin, a condition conducive for longer-term EcN colonization [29]. Streptomycin resistant versions of WT EcN and *Δpks* EcN were mixed in a 1:1 ratio and co-administered to female C57BL/6J mice (n = 10 per group) that had received streptomycin in their drinking water (5 g/L) for 24h before dosing. The fecal recovery of both WT EcN and *Δpks* EcN peaked at 6 hours post-bacteria administration, with 1.1 × 10^10^ and 8.7 × 10^9^ CFU/g feces, respectively, and the recovery of both strains remained relatively stable over the course of the experiment (Fig 5B). The fecal CFU recovery of *Δpks* EcN was indistinguishable from that of the isogenic control strain across all time points, indicating that there was no observable competitive advantage or disadvantage for a strain containing a *Δpks* mutation *in vivo*. Additionally, in conditions conducive to EcN colonization, the *Δpks* deletion did not result in colonization defects.

### Kinetics and strains activity in NHPs

**Fig 6:**
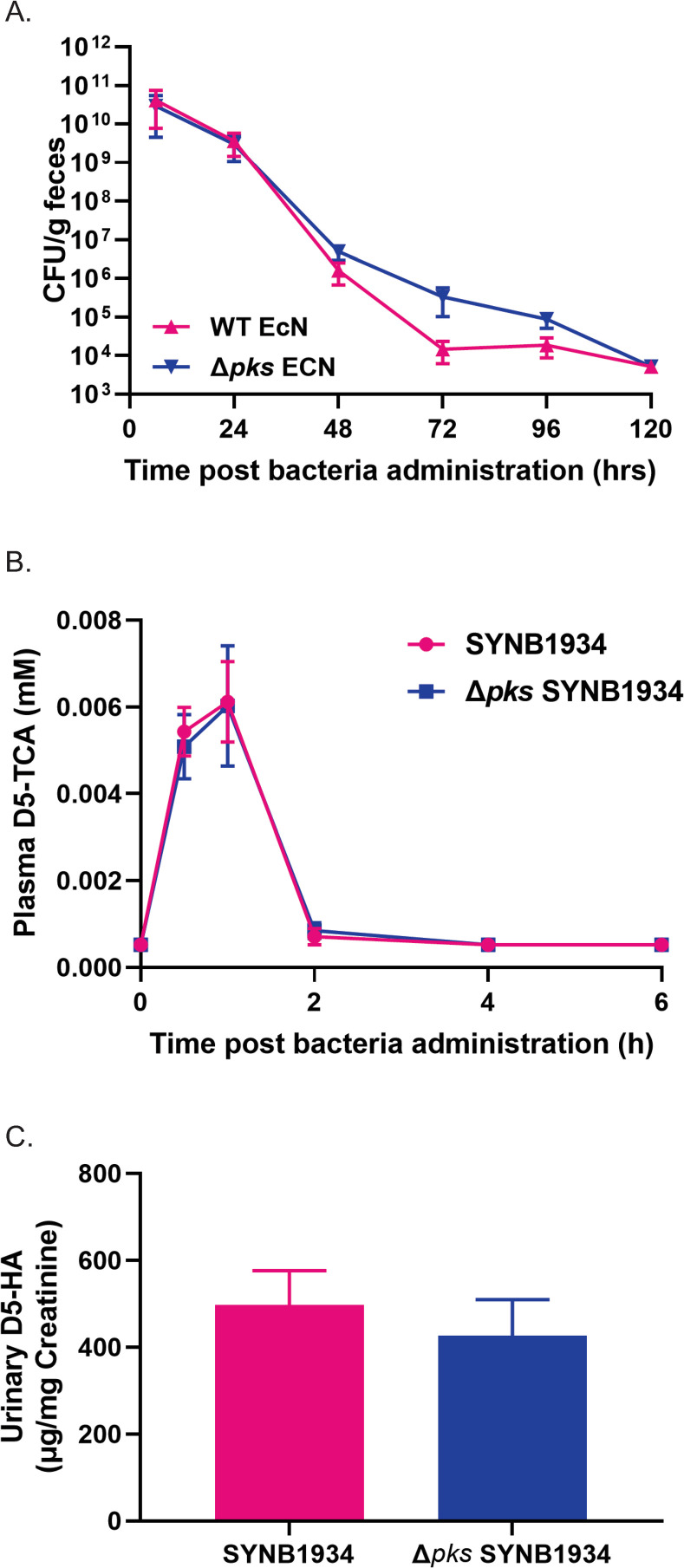
Kinetics and activity of WT EcN and *Δpks* EcN in nonhuman primates (NHPs). (A) Fecal excretion of EcN strains in NHPs (n = 6 per group) following oral administration of a combined dose (1x10^12^ CFU of each bacterial strain) of WT EcN (pink) or *Δpks* EcN (dark blue) over 120h, (LLOQ = 1.5x10^2^ CFU/g feces). Data are presented as mean ± SEM. Statistical analysis was performed using 2-way repeated ANOVA followed by Sidak’s multiple comparison test (p>0.05). (B) Appearance of plasma D5-TCA (B) and urinary recovery of D5-HA (C) in NHPs (n = 12 per group) following oral administration of a single dose (1x10^12^ CFU) of SYNB1934 (pink) or *Δpks* SYNB1934 (dark blue). Data are presented as mean ± SEM. Statistical analysis was performed using 2-way repeated ANOVA followed by Sidak’s multiple comparison test (B) and unpaired t-test with Welch’s correction (C).

To further characterize the potential effects of *pks* deletion on bacterial transit and activity, studies were conducted in NHPs as these animals share a GI physiology that is more relevant to humans. First, transit kinetics and *in vivo* competition were assessed in non-naïve male cynomolgus monkeys (2–5 years of age) who received a single oral dose of a 1:1 combination of WT EcN and *Δpks* EcN at 1x10^12^ CFUs for each strain, with feces collected for up to 120h. Peak fecal excretion of WT EcN and *Δpks* EcN was observed at the first timepoint collected (6h), with 4.1 × 10^10^ and 2.9 × 10^10^ CFU/g feces, respectively, and no significant difference between the amount of each strain recovered was reported for the duration of the experiment (Fig 6A). By 120h, 99.99% of the bacterial strains had been cleared in each group. Overall, these findings demonstrate that both WT EcN and *Δpks* EcN have a similar profile of GI transit and survival in NHPs, consistent with the results observed in mice.

A subsequent NHP study was designed to assess whether the deletion of *pks* in the phenylalanine-consuming strain SYNB1934 has a significant impact on *in vivo* activity. We have previously demonstrated that plasma TCA can be used as a unique and specific biomarker to track PAL activity of engineered strains, and that administration of WT EcN does not result in TCA accumulation [19]. Administration of an oral bolus of d5-Phe, peptone, and SYNB1934 or *Δpks* SYNB1934 (1 x 10^11^ CFUs) resulted in no significant difference in the plasma exposure to d5-TCA, both in terms of the kinetics of TCA appearance in plasma and maximal concentrations observed (Fig 6B).

TCA produced from engineered strains is quantitatively converted in the liver to hippurate (HA) and targeted for urinary excretion [19,30]. Unlike plasma TCA, urinary HA is a common metabolite found in the urine of primates, resulting in high background levels [30,31]. However, the use of oral d5-Phe as a metabolic tracer allows for the calculation of HA excretion attributable to engineered strains because there is no natural route for the conversion of Phe to HA, and thus the only metabolic route from oral d5-Phe to urinary d5-HA is through the d5-TCA intermediate produced in engineered strains by PAL. Similar to what was observed for plasma d5-TCA, there was no significant difference in the urinary recovery of d5-HA between SYNB1934 and *Δpks* SYNB1934 over a 6h period (Fig 6C). Taken together, these results further demonstrate that deletion of *pks* in SYNB1934 has no effect on *in vivo* survival or PAL activity *in vivo*.

## Discussion

To date, several studies have demonstrated an association of CRC with the presence of *pks*^*+*^ [3–5,9]. Despite the suggested direct link between colibactin and CRC, specifically through APC gene mutation [4,5], some questions have yet to be answered, making it difficult to claim a direct correlation between the two. Notably, *pks*^*+*^ bacteria are found in 20% of healthy individuals, indicating that carcinogenesis appears in some individuals but not all. In addition, whether EcN induces a similar mutational signature as other *pks*^*+*^ strains have yet to be investigated. Moreover, *pks* gene regulation and the role environmental factors might play in this context is also not fully understood at the moment. With regards to both EcN and *pks*, the totality of the available data from animals and humans suggests that colibactin could be involved in oncogenesis, but not without some other form of predisposition. It is clear that colibactin can illicit genotoxic effects leading to tumorigenesis in mammalian cells *in vitro*, *ex vivo*, and in specific preclinical mouse models, and therefore reasonable concerns have been raised with regards to the existence of colibactin production by the gut microbiota. These concerns include consideration of the safety of over-the-counter probiotics such as EcN (with an intact *pks* island), which has been used therapeutically by human populations for over 100 years [32]. Additionally, the progression of EcN as a chassis for the development of engineered live bacterial therapeutics, which we have termed Synthetic Biotics, further compounds concern over the presence of *pks*. There have also been reports demonstrating that deletion of the pks island in EcN results in negative impacts on the strain’s probiotic effects. Removal of pks has been reported to lead to a loss in anti-inflammatory, anti-pain, and biological control properties [33–35], which are important considerations in the use of *E*. *coli* Nissle as a probiotic. In this context, it is important to note that Synthetic Biotics do not depend on these properties for therapeutic function. The results presented in this work demonstrate that the removal of *pks* does not alter the transit and clearance kinetics or the engineered therapeutic function of our EcN strains. It is however conceivable that future engineered bacterial therapeutics may be developed to treat diseases that may additionally benefit from the intrinsic probiotic properties of EcN. For such therapeutic programs, further investigation as to the role of colibactin would be warranted. A thorough investigation of potential deleterious roles of colibactin is warranted in light of the fact that such strains offer the potential to be transformative therapeutic options for patients, and several EcN-derived strains have already been advanced to clinical trials [18–20,36].

Synthetic Biotic EcN strains have been engineered as potential treatments for patients with PKU and enteric hyperoxaluria by degrading pathogenic metabolites in the GI tract to prevent their absorption and to reduce systemic exposure. Although the initial strains developed contained the *pks* island, the risk of unintended effects due to colibactin in early clinical trials is low given the short duration of exposure, the lack of strain colonization, and the absence *in vivo* genotoxicity of the parent strain in preclinical studies [11]. It is important to note that EcN has an established record of safe probiotic use for the treatment of various GI conditions, and to date there have been no apparent reported increases in the risk of developing CRC associated with the use of EcN [37–41]. EcN is generally cleared quickly from the human colon without establishing long-term colonization and Synthetic Biotic strains are further replication-deficient in the intestinal tract due to the engineering of nutritional auxotrophies, rendering cells unable to divide without an exogenously added essential compound [42]. Indeed, the recent Nougayrede *et*. *al*. demonstration of the genotoxic potential of EcN was dependent on *in vivo* cell division and colonization [10], which are capabilities removed from Synthetic Biotic strains through genetic engineering.

While the science concerning colibactin and its role in CRC progression is still evolving, risks associated with longer duration exposure to EcN, as well as potential mitigation strategies, must be addressed proactively ahead of future long-term clinical studies using EcN and other colibactin producing bacterial strains. To that end, the work described herein assessed the feasibility of *pks* island removal from EcN and related Synthetic Biotic strains without compromising strain fitness and activity, in anticipation of long-term patient dosing. For the strains used throughout this study, all genes in the *pks* island were removed with the exception of *clbS*, which is reported to confer immunity to colibactin. However, the putative *clbS* promoter region was also removed during *pks* deletion and our transcriptomic data suggested that this gene was not significantly expressed in the *Δpks* background. That being said, we cannot completely rule out the possibility that *clbS* is expressed at a low basal level that could have affected strain performance in these studies. The *clbS* gene product may have additional roles beyond protection from the toxic effects of colibactin, including direct protection of DNA from nucleolytic degradation [43]. Removal of *pks* island (*ΔclbA-clbQ)* from multiple strains of EcN resulted in no observable growth or fitness cost, both *in vitro* and *in vivo*. Furthermore, the *pks* island was dispensable for activity in multiple clinical candidate Synthetic Biotic strains both *in vitro* and *in vivo*. The results described here suggests that an EcN chassis organism deleted for the *pks* island provides an opportunity to further mitigate potential risks associated with colibactin production without having a negative effect on engineered strain activity and efficacy.

## Supporting information

S1 Table Expression level of colibactin producing genes after removal of the *pks* loci. Successful removal of the colibactin production *loci* in *Δpks* SYNB8802 prevents expression of *clbS* gene responsible for colibactin resistance (in red). *clbS* expression is retained in SYNB8802.
(XLSX)

S1 Fig *In vitro* evaluation of EcN and *Δpks* EcN in minimal media (M9). Growth curves of A) WT EcN, B) *Δpks* EcN and C) both strains in mixed culture/competition (inoculated at a 1:1 ratio) grown in minimal media (M9) + three concentrations of FeSO4, 0μM (blue), 100 μM(orange), 200 μM (gray). Data are the mean and SD of triplicate cultures.
(TIF)
