# Supplementary figures and images for: Robust performance of a live bacterial therapeutic chassis lacking the colibactin gene cluster
Source: PLoS One. 2023 Feb 2;18(2):e0280499. doi: 10.1371/journal.pone.0280499 (PMC9894410; doi:10.1371/journal.pone.0280499)

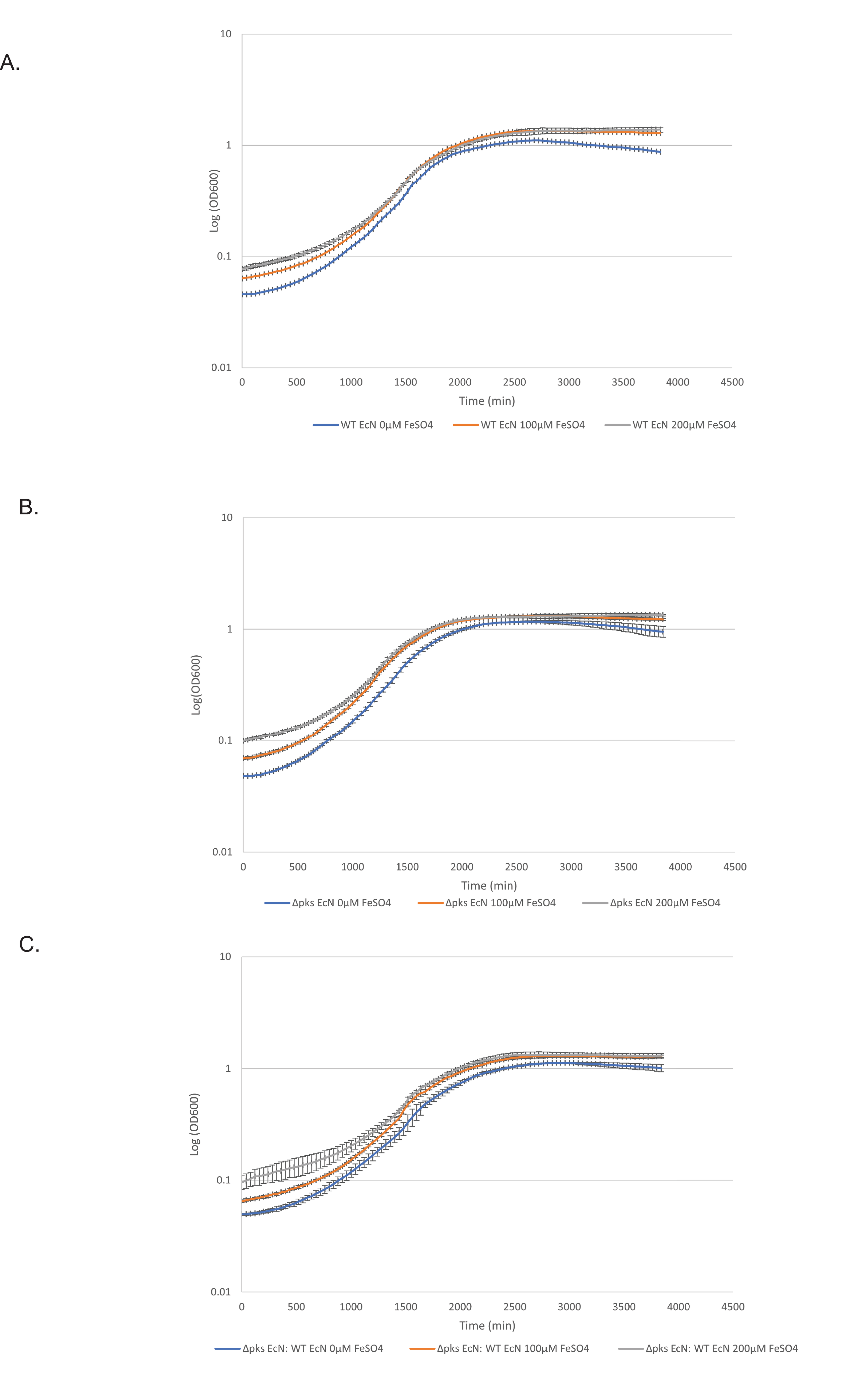

Supplement: S1 Fig — Growth curves of A) WT EcN, B) Δpks EcN and C) both strains in mixed culture/competition (inoculated at a 1:1 ratio) grown in minimal media (M9) + three concentrations of FeSO4, 0μM (blue), 100 μM(orange), 200 μM (gray). Data are the mean and SD of triplicate cultures. (TIF) [file pone.0280499.s002.tif]
